# Platelet adhesion in type 2 diabetes: impact of plasma albumin and mean platelet volume

**DOI:** 10.1186/s12959-021-00291-w

**Published:** 2021-06-02

**Authors:** Mona Johansson, Andreas C. Eriksson, Carl Johan Östgren, Per A. Whiss

**Affiliations:** 1 Department of Biomedical and Clinical Sciences (BKV), Division of Clinical Chemistry and Pharmacology, Linköping University, Building 420, Entrance 68, Level 8, Campus US, SE-581 83 Linköping, Sweden; 2 Department of Health, Medicine and Caring Sciences (HMV), Division of Prevention, Rehabilitation and Community Medicine, Linköping University, SE-581 83 Linköping, Sweden

**Keywords:** Type 2 diabetes, Platelet adhesion, Mean Platelet Volume, Plasma magnesium, Plasma albumin

## Abstract

**Background:**

Altered mean platelet volume (MPV) and plasma albumin has been reported in type 2 diabetes (T2D). MPV is suggested to predict cardiovascular risk but there is a lack of evidence for associations between MPV and platelet adhesion. Plasma albumin and magnesium are other factors reported to influence thrombotic risk. The objectives of this study were to assess the association between platelet adhesion and plasma factors with a potential role to affect platelet activation.

**Methods:**

Blood was collected from 60 T2D patients and 60 healthy controls. Platelet adhesion to different protein surfaces induced by various soluble activators were measured in microplates. MPV, albumin and magnesium were analysed together with additional routine tests.

**Results:**

Despite normal levels, plasma albumin significantly correlated with adhesion of T2D platelets but not with controls. There was a significant association between MPV and platelet adhesion in both groups, but association was smaller in T2D. Levels of glucose, HbA1c or magnesium did not correlate with platelet adhesion.

**Conclusions:**

Plasma albumin was associated with platelet adhesion in T2D suggesting that albumin may be a factor to consider upon cardiovascular risk assessment. MPV was more associated with the level of platelet adhesion in healthy individuals than in well-controlled T2D patients.

## Introduction

Platelets play a crucial role in hemostasis with adhesion, aggregation and pro-coagulative function upon tissue damage. When injuries or inflammatory reactions affect the endothelial layer in blood vessels, platelets become activated and adhere to exposed subendothelial proteins such as collagen [1]. Activated platelets secret granules with compounds such as fibrinogen, adenosine diphosphate (ADP) and calcium necessary for the normal hemostasis but platelets are also involved in development of atherosclerosis and subsequent thrombus formation.

Patients with Type 2 diabetes (T2D) evidently have increased risk for cardiovascular disease and thrombotic complications compared to controls [2]. Numerous physiological changes that affect hemostasis have been reported in diabetes. Among these, platelets in diabetes have been reported to be characterised by alterations such as increased aggregation or other activation dependent events [1, 3].

Although several studies have reported that the size of platelets does matter, the clinical use of platelet indices is presently unclear [4–6]. Mean platelet volume (MPV) is reported to be significantly increased in subjects with arterial thrombotic events and an elevated MPV is also associated with poor outcome. Higher MPV has been reported in patients with T2D as compared to healthy individuals in some but not all studies [7]. Furthermore, diabetic patients with higher levels of HbA1c have been reported to have higher levels of MPV [8, 9], and improved glycemic control has been found to normalize both MPV and HbA1c levels [10, 11]. Furthermore, several findings suggest that women with gestational diabetes may be accompanied by increased MPV levels [12]. Increased levels of MPV have also been found to associate with the development of retinopathty in patients with T2D [13] suggesting that MPV can be used as a marker of microvascular complications. However, in a previous study MPV was not related to prevalence or extent of coronary artery disease in diabetic patients [14]. The direct relationship between MPV, platelet activation and diabetes are poorly examined. Earlier studies have also reported absence of relationship between MPV and platelet aggregation measured in platelet rich plasma in healthy individuals [15] as well as in diabetic patients [14]. Platelet distribution width (PDW) is another measure of platelets that has been associated with carotid intima-media thickness and vascular complications in diabetes [16, 17].

Magnesium and albumin are examples of several factors that have been suggested to play a role in increased platelet activity and trombotic risk in diabetes. Magnesium ions (Mg^2+^) is an important factor for platelet adhesion [18, 19] as well as plasma coagulation [3]. Several studies have also investigated the association of serum or plasma magnesium with prediabetes and T2D. The findings are inconsistent, but some studies show that low blood level of magnesium is a strong independent predictor of incident T2D [20, 21]. The major plasma protein albumin can influence platelet function as well as atherogenesis and thrombosis in diabetes and other conditions [22]. A recent meta-analysis concluded that there is a robust and independent relationship with low plasma albumin and cardiovascular disease events [23]. Earlier reported effects of albumin on platelets are multiple and varying but a suggested role is to antagonise several prothrombotic actions and an anticoagulant action is also reported [24].

Despite that platelet adhesion is one of the earliest events upon platelet activation, this function is rarely studied with intention to understand thrombotic events and correlation with clinically used analytes or outcome measures. Since the impact of MPV, albumin and magnesium on platelet adhesion are not earlier studied in human T2D subjects, the aim of the current study was to investigate how these factors associate with platelet adhesion in type 2 diabetes.

## Materials and methods

### Subject Population and Blood Sampling

T2D patients in this study were participating in the first phase of a community-based cohort study, Cardiovascular Risk factors in Patients with Diabetes — a Prospective study in Primary care (CARDIPP-1) [25]. The general aim of this study was to explore the prevalence and impact of cardiovascular risk factors in patients with T2D, aged 55–65 years. The patients were recruited from 7 different primary healthcare centres in the county of Jönköping, Sweden.

Venous blood from 60 patients with T2D was collected when subjects were in a nonfasting state at the Department of Physiology at County Hospital Ryhov, Jönköping, Sweden. Blood was collected in two 7 mL sodium heparin tubes (Becton Dickinson, Oxford, UK) for platelet count, characteristics and adhesion, as well as magnesium and albumin analysis; 3 mL in one EDTA tube for HbA1c and; 4 mL in one sodium fluoride + oxalate tube (Becton Dickinson) for plasma glucose (P-glucose). The same quantity of venous blood was collected from 60 age and sex-matched healthy blood donors to serve as controls. Only donors declaring that they had not used any drugs known to interfere with platelet function the previous 14 days was included in the study. Participants did also declare that they refrained from using tobacco and caffeine 3 h prior to the blood sampling as well as alcohol 12 h prior to the sampling.

### Routine Biochemical Determinations

After blood collection and with similar time delays between sampling and analysis, routine laboratory blood count and characteristics were determined by Cell-Dyn Hematology Analyzer (Abbott Diagnostics, Abbott Park, IL, USA). For measuring magnesium, albumin and glucose, blood was centrifuged for 5 min at 1000 x g and were then analysed utilising the clinical chemistry analyzer Advia 1650 (Siemens, Tarrytown, NY, USA) at the Department of Clinical Chemistry, Ryhov, Jönköping, Sweden. HbA1c was analyzed with a High-Pressure Liquid Chromatography technique (Mono S, Farmacia, Uppsala, Sweden).

### Preparation of platelet rich plasma

After 20 min in room temperature, 7 mL blood was centrifuged for 20 min at 205 x g. The platelet rich plasma (PRP) was collected, and platelets were thereafter counted in the CellDyn Hematology Analyzer. PRP was transferred to a new plastic tube and diluted 1:4 in a solution of 0,9 % NaCl containing 5 mmol/L MgCl_2_.

### Coating of microplates

Coating of 96-well microplates (Nunc Maxisorp, Roskilde, Denmark) were made by addition of 100 µL of four different protein solutions followed by incubation at 5° C for 1–2 days, allowing adsorption to the wells [19]. The protein solutions consisted of 2 mg/mL human albumin (Pharmacia & Upjohn AB, Stockholm, Sweden), 2 mg/mL human fibrinogen (human Grade L, American Diagnostica Inc., Stamford, CT, USA) or 0,1 mg/mL bovine collagen (Roche Diagnostics, Oxford, UK).

### Platelet adhesion assay

Static platelet adhesion was measured by an assay as described earlier [19, 26]. Protein-coated microplates were manually washed twice in 0.9 % NaCl by plate inversion. Then 50 µL of four different platelet-activating substances in various concentrations were added to the wells followed by the addition of 50 µL diluted PRP. The platelet activators were ADP (Sigma-Aldrich, St Louis, Missouri, USA), adrenalin (Merck NM AB, Stockholm, Sweden) and ristocetin sulfate (Sigma-Aldrich). The platelets were then allowed to adhere to the well surfaces shaking gentle during one hour in room temperature. Unbound platelets were then removed by washing twice in 0.9 % NaCl by plate inversion. Directly, 140 µL of a substrate solution, p-nitrophenyl-phosphate (1 mg/mL; Sigma-Aldrich) dissolved in buffer (pH 5,4) containing 0.1 mol/L sodium citrate, 0.1 mol/L citric acid and 0.1 % Triton X-100 was added to each well. This incubation allows the occurrence of an enzymatic reaction between p-nitrophenyl-phosphate and platelet acid phosphatase which result in a soluble product. A separate microplate was used to get an estimated value for 0 and 100 % platelet attachment for each subject. This was achieved by mixing 50 µL 0.9 % NaCl (0 %) and 50 µL diluted PRP (100 %) with 140 µL substrate solution. Background absorbance was measured for all wells at 405 nm using an Emax precision microplate reader (Molecular Devices, Sunnyvale, CA, USA). Microplates were then incubated for 40 min during constant shaking at room temperature. After incubation, 100 µL of 2 mol/L NaOH was added to end the enzymatic reaction, followed by absorbance measurement at 405 nm. This was performed for all microplates, including the microplates for measuring 0 and 100 % attachment. Percentage adhesion of platelets was calculated subsequently according to the absorbance values.

### Statistical analysis

Comparison of baseline characteristics between groups was based on two-tailed unpaired *t*-test (parametric distribution) with Welch’s correction. For platelet adhesion studies, PRP were prepared from the stated number of patients or controls for each experiment. The mean of quadruplicates was used for calculations. The effects of different protein surfaces and externally added compounds on adhesion within-group was analysed with one-way repeated measures ANOVA and two-tailed Mann-Whitney test (nonparametric) was used to compare platelet adhesion between groups. Correlation between factors were investigated with Pearson’s correlation coefficients, two-tailed 95 % confidence interval. A *p* value of < 0.05 was judged as statistically significant. GraphPad Prism® was used for statistical analysis (version 8.4.3; GraphPad Software Inc, San Diego, Ca).

## Results

### Demographical and laboratory data

The T2D patients showed significantly higher (*p* < 0.001) P-glucose and HbA1c levels (10.1 ± 4.2 mmol/L and 58.6 ± 16.2 mmol/mol) as compared to healthy controls (5.7 ± 1.1 mmol/L and 36.5 ± 7.1 mmol/mol). HbA1c was significantly correlated with P-glucose in T2D patients (r = 0.479; *p* = 0.013) but not in healthy controls (r = 0.282; *p* = 0.139). Additional characteristics of healthy controls and T2D patients as well as medical therapy for the patients are shown in Table 1.

**Table 1:** Healthy control and patient characteristics

Demographical and laboratory data	Healthy controls (n = 60)	Type 2 diabetic patients (n = 60)
Female/Male sex (n)	28/32	28/32
Age, years (mean ± SD)	60.1 ± 3.5	60.8 ± 3.5
Platelet count, 10^9^/L (mean ± SD)	210.5 ± 103.3	200.1 ± 83.7
P-Magnesium, mmol/L (mean ± SD)	0.85 ± 0.07	0.82 ± 0.08
P-Albumin, g/L (mean ± SD)	41.6 ± 2.9	41.0 ± 3.7
***Therapy at admission (n)***		
Insulin	-	19
Peroral antidiabetics	-	37
Acetylsalicylic acid (ASA)	-	16
Anticoagulants or Platelet inhibitor (other than ASA)	-	2
Anti-inflammatory drugs	-	3
ACE inhibitors	-	16
Angiotensin receptor blockers (ARB)	-	5
Beta blockers	-	16
Calcium antagonists	-	7
Diuretics	-	14
Statins	-	29
Selective serotonin reuptake inhibitors (SSRI)	-	4
Bensodiazepines	-	1

### Platelet adhesion

**Fig. 1:**
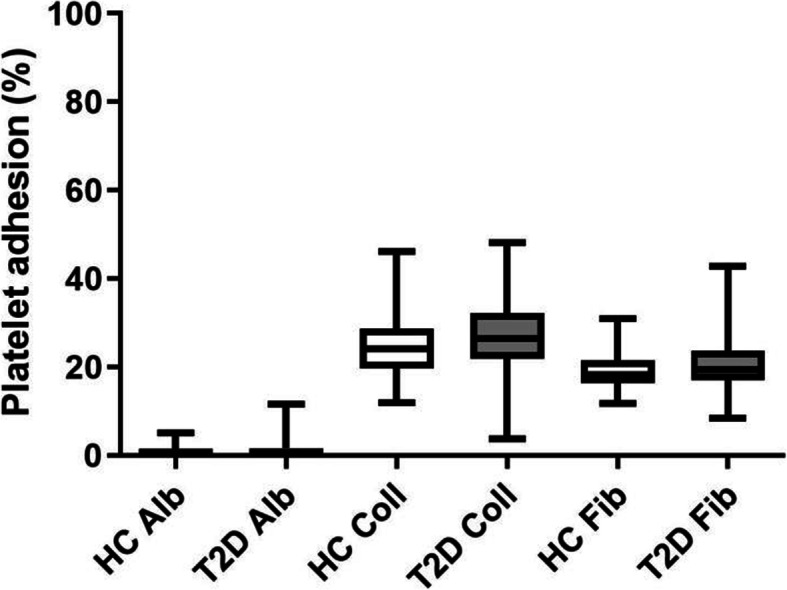
In both healthy controls (HC, *n* = 60) and Type 2 diabetic subjects (T2D, *n* = 60), the collagen (Coll) and fibrinogen (Fib) surfaces caused significantly more platelet adhesion as compared to albumin (Alb), and the collagen surface caused significantly more adhesion than fibrinogen ( *p* < 0.001, for all comparisons). There was no significant difference in adhesion upon comparison between controls and T2D subjects. The box shows 25th to 75th percentiles and whiskers show the minimum to the maximum value of all points. The middle line in the box shows the median

In both healthy controls and T2D subjects, the collagen and fibrinogen surfaces caused significantly more platelet adhesion as compared to the albumin surface. The collagen surface caused significantly more adhesion than fibrinogen (*p* < 0.001, for all comparisons). However, there were no differences in adhesion between healthy controls and T2D subjects (Fig. 1).

**Fig. 2:**
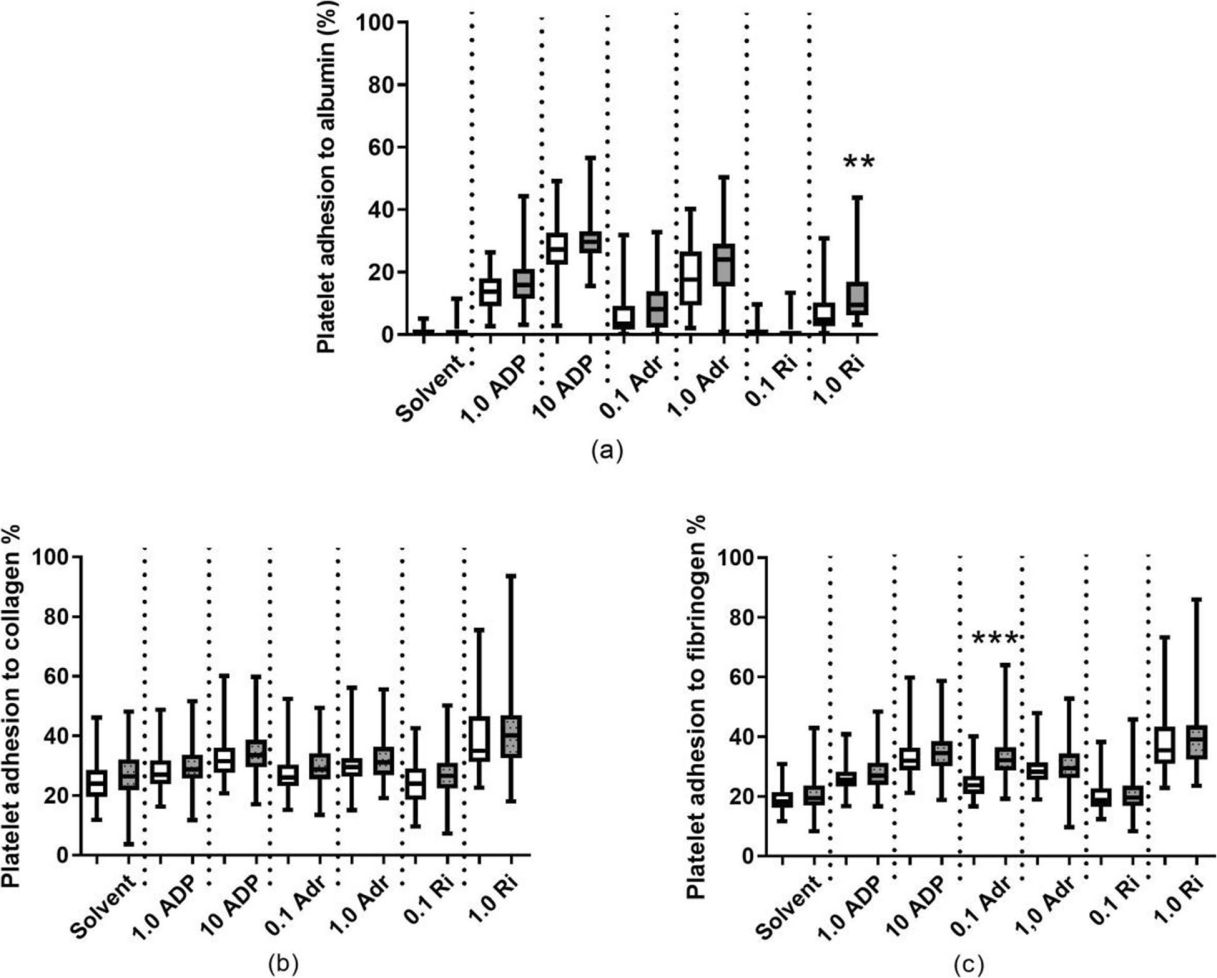
Adhesion of platelets from healthy controls (white, *n* = 60) and type 2 diabetic patients (grey, *n* = 60) to (**a**) albumin; (**b**) collagen; (**c**) fibrinogen upon activation with 1.0 and 10 µmol/L adenosine diphosphate (ADP); 0.1 and 1.0 µmol/L Adrenaline (Adr); or 0.1 and 1.0 mg/mL Ristocetin (Ri). Solvent denotes basal adhesion to protein surface without any soluble activator. The box shows 25th to 75th percentiles and whiskers show the minimum to the maximum value of all points. The middle line in the box shows the median. **p < 0.01 and ***p < 0.001 between type 2 diabetic patients and healthy controls

Except for 0.1 µmol/L ADP and 0.1 mg/mL ristocetin, all tested concentrations of activators significantly increased adhesion of both control and T2D platelets to albumin (*p* < 0.0001, Fig. 2a). Upon activation with 1.0 mg/mL ristocetin, adhesion of platelets from T2D subjects was significantly increased as compared to control platelets (*p* < 0.01). On the collagen surface, the highest concentrations of ADP (10 µmol/L), adrenaline (1 µmol/L), and ristocetin (1,0 mg/mL) increased platelet adhesion as compared to solvent (*p* < 0.001). The results were similar for platelets from controls and T2D patients (Fig. 2b). Except for 0.1 mg/mL ristocetin, all tested compounds increased adhesion of both control and T2D platelets to fibrinogen (*p* < 0.001). At 0.1 µmol/L adrenaline, platelets from T2D patients were significantly more prone to adhere (*p* < 0.001). The median value for adhesion on fibrinogen induced by 0.1 µmol/L adrenaline was 33 % for platelets from T2D patients and 24 % for control platelets (Fig. 2c).

### Blood characteristics and platelet adhesion

**Fig. 3:**
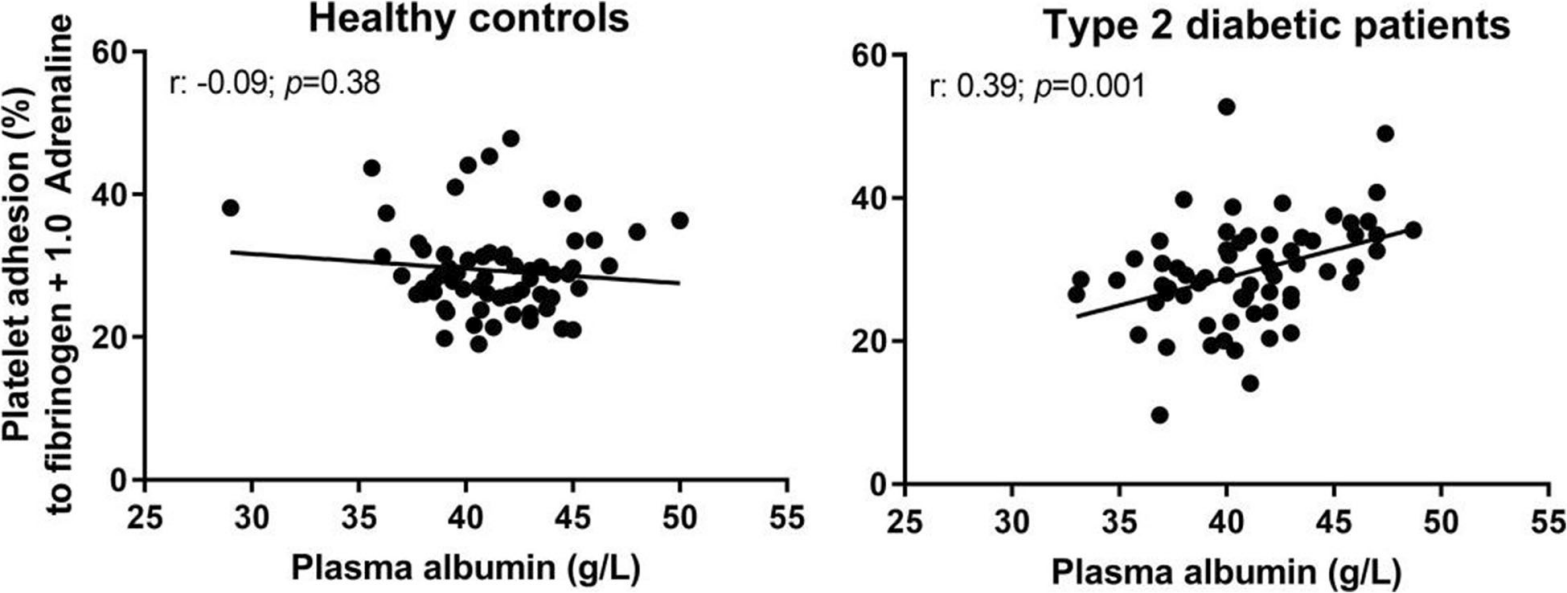
Plasma albumin (g/L) scatter-plotted against per cent platelet adhesion to fibrinogen in presence of 1.0 µmol/L adrenaline (healthy controls *n* = 60; type 2 diabetic patients *n* = 60)

The levels of plasma magnesium (Table 1) were not correlated to platelet adhesion. However, the levels of plasma albumin did positively correlate with most adhesion conditions in T2D patients but not in healthy controls (Table 2 for all comparisons and Fig. 3 for representative example). Blood levels of glucose and HbA1c did not correlate with adhesion of platelets from any group.

**Table 2:** Relationship between plasma albumin and platelet adhesion at different conditions by Pearson correlation coefficients (Pearson r, *P* < 0.05 bold*)*. Albumin surface (Alb); adenosine diphosphate (ADP); Adrenaline (Adr); Ristocetin (Ri); Collagen surface (Coll); Fibrinogen surface (Fib)

Surface + concentration of soluble activator	Healthy controls (*n* = 60)		Type 2 diabetic patients (*n* = 60)	
	Pearson r	*P* value	Pearson r	*P* value
Alb	-0,048	*0,416*	-0,022	*0,862*
Alb + 0.1 µmol/L ADP	-0,06	*0,461*	0,012	*0,923*
Alb + 1.0 µmol/L ADP	-0,037	*0,970*	0,159	*0,197*
Alb + 10 µmol/L ADP	-0,145	*0,837*	**0,286**	***0,018***
Alb + 0.1 µmol/L Adr	-0,037	*0,357*	0,211	*0,089*
Alb + 1.0 µmol/L Adr	-0,066	*0,187*	0,240	*0,052*
Alb + 0,1 mg/mL Ri	-0,057	*0,364*	0,048	*0,696*
Alb + 1,0 mg/mL Ri	0,084	*0,382*	0,120	*0,334*
Coll	0,022	*0,353*	**0,316**	***0,009***
Coll + 0.1 µmol/L ADP	-0,055	*0,316*	**0,300**	***0,014***
Coll + 1.0 µmol/L ADP	-0,092	*0,567*	**0,329**	***0,006***
Coll + 10 µmol/L ADP	-0,124	*0,919*	**0,324**	***0,007***
Coll + 0.1 µmol/L Adr	-0,090	*0,364*	**0,335**	***0,005***
Coll + 1.0 µmol/L Adr	-0,083	*0,409*	**0,314**	***0,009***
Coll + 0,1 mg/mL Ri	-0,046	*0,217*	**0,296**	***0,020***
Coll + 1,0 mg/mL Ri	-0,240	*0,206*	0,199	*0,106*
Fib	-0,177	*0,794*	**0,301**	***0,013***
Fib + 0.1 µmol/L ADP	-0,203	*0,537*	**0,317**	***0,009***
Fib + 1.0 µmol/L ADP	-0,138	*0,441*	**0,356**	***0,003***
Fib + 10 µmol/L ADP	-0,070	*0,872*	**0,358**	***0,003***
Fib + 0.1 µmol/L Adr	-0,089	*0,353*	0,071	*0,566*
Fib + 1.0 µmol/L Adr	-0,090	*0,377*	**0,390**	***0,001***
Fib + 0,1 mg/mL Ri	-0,222	*0,920*	**0,329**	***0,009***
Fib + 1,0 mg/mL Ri	-0,191	*0,532*	0,221	*0,072*

### Platelet characteristics

Mean platelet volume (MPV) and platelet deviation width (PDW) were determined in both whole blood as well as the PRP that was used in the adhesion assay. For healthy controls, the MPV in whole blood was 7.6 ± 0.8 fL (mean ± SD) and 6.3 ± 0.6 fL in PRP (*p* < 0.001). The corresponding values for MPV in T2D were 7.8 ± 0.8 in whole blood and 6.5 ± 0.7 fL in PRP (*p* < 0.001). Thus, MPV decreased by 17 % during the preparation of PRP from whole blood in both healthy as well as T2D patients. PDW was 15.9 ± 0.7 respectively 15.2 ± 1.1 in whole blood and PRP from healthy controls (*p* < 0.05) and the corresponding values in T2D patients were 16.0 ± 0.5 in whole blood and 15.4 ± 1.1 in PRP (*p* < 0.01). Accordingly, PDW was approximately 4 % lower in PRP as compared to whole blood in both groups. Taken together, both MPV and PDW were significantly lower in PRP as compared to whole blood but there were no significant differences between T2D patients and healthy individuals. MPV or PDW did not correlate with P-glucose or HbA1c in any group. Plateletcrit (PCT) was only determined in whole blood and was 0.17 ± 0.1 in controls and 0.17 ± 0.1 in T2D patients.

**Fig. 4:**
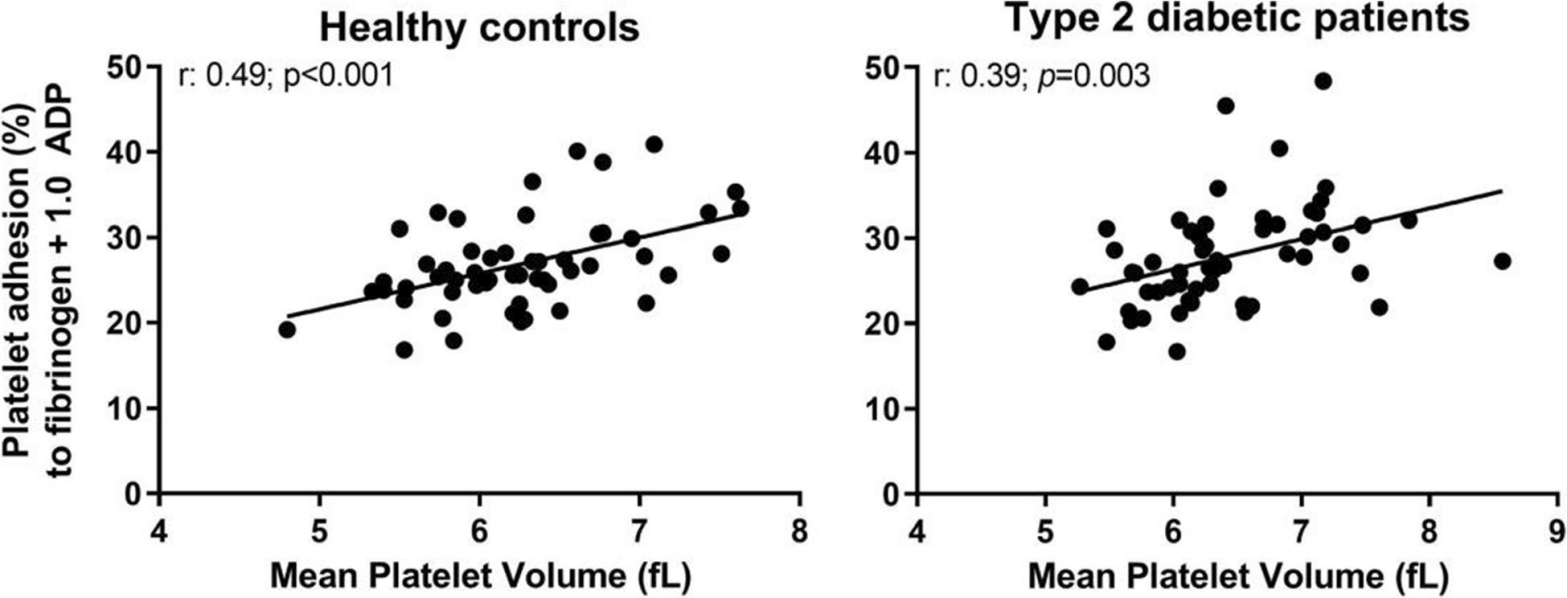
Mean Platelet Volume (fL) scatter-plotted against per cent platelet adhesion to fibrinogen in presence of 1.0 µmol/L ADP (healthy controls *n* = 60; type 2 diabetic patients *n* = 60)

MPV was significantly correlated to platelet adhesion on all surfaces induced by ADP and adrenaline, but MPV did not correlate to basal adhesion on albumin or to ristocetin-induced adhesion on any surface. The correlation coefficient (Pearson r) ranged from 0.25 to 0.49 in healthy controls and from 0.26 to 0.41 in T2D patients for the different surfaces and soluble activators (Table 3 for all comparisons and Fig. 4 for example). Control platelets showed higher correlation, compared to platelets from T2D patients, in 15 out of 18 conditions where adhesion and MPV were related. This indicates that the correlation of MPV with adhesion was more pronounced in healthy controls compared to T2D patients. PDW did not correlate with platelet adhesion to any surface or treatment.

**Table 3:** Relationship between mean platelet volume (MPV) and platelet adhesion at different conditions by Pearson correlation coefficients (Pearson r, P < 0.05 bold). Albumin surface (Alb); adenosine diphosphate (ADP); Adrenaline (Adr); Ristocetin (Ri); Collagen surface (Coll); Fibrinogen surface (Fib)

Surface + concentration of soluble activator	Healthy controls (n = 60)		Type 2 diabetic patients (n = 60)	
	Pearson r	*P* value	Pearson r	*P* value
Alb	-0,026	*0,853*	0,085	*0,531*
Alb + 0.1 µmol/L ADP	0,249	*0,073*	0,213	*0,115*
Alb + 1.0 µmol/L ADP	**0,414**	***0,002***	**0,413**	***0,002***
Alb + 10 µmol/L ADP	**0,493**	***< 0,001***	**0,264**	***0.049***
Alb + 0.1 µmol/L Adr	0,248	*0,073*	**0,311**	***0,021***
Alb + 1.0 µmol/L Adr	**0,488**	***< 0,001***	**0,270**	***0.046***
Alb + 0,1 mg/mL Ri	-0,093	*0,571*	0,031	*0,820*
Alb + 1,0 mg/mL Ri	0,039	*0,778*	0,024	*0,861*
Coll	**0,417**	***0,002***	**0,395**	***0,003***
Coll + 0.1 µmol/L ADP	**0,451**	***< 0,001***	**0,390**	***0,003***
Coll + 1.0 µmol/L ADP	**0,444**	***< 0,001***	**0,385**	***0,003***
Coll + 10 µmol/L ADP	**0,327**	***0,016***	**0,333**	***0,012***
Coll + 0.1 µmol/L Adr	**0,418**	***0,002***	**0,371**	***0,005***
Coll + 1.0 µmol/L Adr	**0,413**	***0,002***	**0,346**	***0,009***
Coll + 0,1 mg/mL Ri	**0,434**	***0,005***	**0,285**	***0.042***
Coll + 1,0 mg/mL Ri	0,186	*0,179*	0,111	*0,415*
Fib	**0,338**	***0,013***	**0,330**	***0,013***
Fib + 0.1 µmol/L ADP	**0,433**	***0,001***	**0,344**	***0,009***
Fib + 1.0 µmol/L ADP	**0,492**	***< 0,001***	**0,394**	***0,003***
Fib + 10 µmol/L ADP	**0,384**	***0,004***	**0,356**	***0,007***
Fib + 0.1 µmol/L Adr	**0,389**	***0,004***	-0,033	***0.081***
Fib + 1.0 µmol/L Adr	**0,426**	***0,001***	**0,419**	***0,001***
Fib + 0,1 mg/mL Ri	0,214	0,183	**0,355**	***0,010***
Fib + 1,0 mg/mL Ri	0,218	*0,116*	0,183	*0,176*

## Discussion

Even if platelet adhesion is established as an initial and critical process in both hemostasis and thrombosis, adhesion is an aspect of platelet function in diabetes that has not been investigated much in earlier studies. The static platelet adhesion assay used in the present study [19, 26] offers opportunities to simultaneously measure combined effects of several different soluble activators and protein surfaces on platelet adhesion and activation.

With various surfaces and soluble activators, there were no difference in basal adhesion to the different surfaces upon comparison between controls and T2D subjects in the present study. This result agrees with few earlier studies on adhesion of platelets from diabetic patients using different preparations and assays [27, 28].

Platelet aggregation is more frequently studied than adhesion in T2D. Some studies report increased aggregation of platelets from T2D patients upon activation with ADP [29, 30]. Increased aggregation upon activation with adrenaline or collagen in T2D has been reported in some studies [30] but not in others [29]. Platelet aggregation has been reported to not correlate with the levels of HbA1c in T2D patients [29] whereas especially adrenaline-induced aggregation is reported to be increased in patients with suboptimal glycemic control compared to patients with optimal glycemic control [30]. Platelet aggregation response to adrenaline has also been reported to correlate with the diabetes duration [29]. In the present study, adrenaline-induced adhesion on fibrinogen was one of the two conditions that was increased in reasonably well-controlled T2D patients as compared to healthy control platelets.

In the present study we show significant impact of plasma albumin on adhesion of platelets from patients with T2D. Within the nordic reference range of albumin (36–45 g/L) [31], the levels were positively correlated to platelet adhesion to collagen and fibrinogen. This correlation was not evident with platelets from healthy individuals. Earlier reported impact of albumin on platelets are multiple and varying, but a suggested role is that albumin antagonise several prothrombotic actions [22]. An anticoagulant action of albumin is also reported [24]. There are however no previous studies reporting that albumin can increase platelet adhesion. The results in the present study of albumin association to increased adhesion solely in T2D patients can be an effect of an impaired regulating effect of albumin on platelets from diabetes patients. Increased susceptibility of platelet activation in diabetes has been suggested in several studies, but albumin is rarely mentioned. However, the presence of advanced glycation end products (AGEs) is one theory on how cardiovascular pathologies are initiated in diabetes [32]. High blood glucose cause glycation of proteins such as hemoglobin (leading to increased HbA1c) as well as albumin and it has been shown that glycated albumin enhances surface receptor expression and aggregation of platelets [33]. In a study on a “relatively healthy Korean population”, higher levels of serum albumin did associate with obesity, high blood pressure and fasting glucose levels, as well as atherogenic dyslipemic profile and insulin resistance [34]. The finding of a positive association of albumin with platelet adhesion in the present study is important in the view of an earlier study reporting a positive and independent association between serum albumin and incident T2D risk [35], but another study have reported inverse associations [36]. In addition, decreased plasma albumin concentration in older persons (58–88 years), even within the normal range, have been reported to increase the risk of incident cardiovascular disease [37], indicating that this predominant blood protein has a fundamental and complex role in the process of thrombosis. Furthermore, the most recent report in this area concluded that there is a relationship with low plasma albumin and cardiovascular disease [23].

In the present study, MPV was positively correlated with adhesion of platelets from both healthy individuals and T2D patients, but the correlation was more pronounced in healthy individuals. Association of MPV with platelet adhesion is earlier reported. Polanowska-Grabowska et al. [38] showed in a flow model that the most large/dense platelets exhibited faster adhesion to immobilized collagen whereas there was no significant difference in size-dependent aggregation induced by high doses of ADP or collagen. Reasonably, larger platelets have also been reported to synthesize more thromboxane B2 and bind more fibrinogen and vWf than small platelets [39]. Impact of MPV is however not observed regarding platelet aggregation in additional studies. Beyan et al. [15] reported that there is no correlation between MPV and ADP-, collagen- or adrenaline-induced platelet aggregation measured in platelet rich plasma in healthy individuals. In accordance, De Luca et al. [14] reported that MPV was not related to collagen-induced platelet aggregation, P-selectin expression, or plasma concentrations of thromboxane B2.

In the present study the MPV and PDW did not differ between healthy controls and T2D patients. However, higher levels of MPV in T2D has been reported in some [10], but not all studies [7]. Higher levels of both MPV and PDW have been reported in T2D patients with increased levels of HbA1c [8], and vascular complications compared to patients without complications [16, 17]. Difference between results of MPV and PDW in different studies can probably be explained using different anticoagulants, measurement times and instrumentation [40]. There was no correlation between MPV and HbA1c values in the present study in accordance with an earlier report [10]. The associations of platelet indices such as MPV and PDW with thrombotic events are not necessarily the cause of thrombotic risk or events but instead the consequence of vascular disease.

Upon activation with the highest concentration of ristocetin in the present study, neither MPV nor albumin did correlate to platelet adhesion in any condition. This difference can probably be explained by the fact that ristocetin induce von Willebrand factor (vWf) dependent platelet adhesion [41], which is a different mechanism than adhesion induced by ADP and adrenaline. Furthermore, whereas adhesion to collagen and fibrinogen independently of soluble activators in the current assay, is produced by α_2_β_1_- respectively α_IIb_β_3_-receptors, ristocetin-induced adhesion is dependent on GPIb-IX-V and von Willebrand factor [26]. In concordance with this, an earlier study did also report that MPV was correlated to collagen-induced, but not ristocetin-induced platelet aggregation [42].

Upon activation with 1.0 mg/mL ristocetin, adhesion of platelets from T2D subjects to albumin was significantly increased as compared to control platelets in the present study. The levels of vWf were not measured in the present study but earlier findings support associations between platelet activation, vWf and T2D. Higher levels of vWf has been reported to associate with increased risk of cardiovascular disease in patients with T2D or insulin resistance [43], and older T2D patients are found to have increased levels of total active vWf compared to younger patients and controls [44].

## Conclusions

The present study shows that MPV are associated with the level of platelet adhesion in both healthy controls and in reasonably well-controlled T2D patients. The plasma concentration of albumin, in normal range, are associated with the levels of platelet adhesion only in T2D. This implicates that plasma albumin may be an additional factor to consider upon cardiovascular risk assessment in T2D and motivates further studies on possible effects of glycation of albumin and the impact on platelet activation.

## Data Availability

Data and statistical analysis are available upon request to corresponding author.

## Abbreviations

ADP: Adenosine diphosphate
HbA1c: Hemoglobin A1c
MPV: Mean platelet volume
PDW: Platelet distribution width
PRP: Platelet rich plasma
T2D: Type 2 diabetes
vWf: Von Willebrand factor
